# Heart Disease and Left Ventricular Rotation – A Systematic Review and Quantitative Summary

**DOI:** 10.1186/1471-2261-12-46

**Published:** 2012-06-24

**Authors:** Aaron A Phillips, Anita T Cote, Shannon SD Bredin, Darren ER Warburton

**Affiliations:** 1 Experimental Medicine Program, Faculty of Medicine, University of British Columbia, Vancouver, Canada; 2 Physical Activity and Chronic Disease Prevention Unit, University of British Columbia, Vancouver, Canada; 3 Rm. 205, Unit II Osborne Centre, 6108 Thunderbird Blvd, University of British Columbia, Vancouver, BC, V6T 1Z3, Canada

**Keywords:** Systematic review, Ventricular twist, Ventricular torsion, Heart disease

## Abstract

**Background:**

Left ventricular (LV) rotation is increasingly examined in those with heart disease. The available evidence measuring LV rotation in those with heart diseases has not been systematically reviewed.

**Methods:**

To review systematically the evidence measuring LV rotational changes in various heart diseases compared to healthy controls, literature searches were conducted for appropriate articles using several electronic databases (e.g., MEDLINE, EMBASE). All randomized-controlled trials, prospective cohort and case–controlled studies that assessed LV rotation in relation to various heart conditions were included. Three independent reviewers evaluated each investigation’s quality using validated scales. Results were tabulated and levels of evidence assigned.

**Results:**

A total of 1,782 studies were found through the systematic literature search. Upon review of the articles, 47 were included. The articles were separated into those investigating changes in LV rotation in participants with: aortic stenosis, myocardial infarction, hypertrophic cardiomyopathy, dilated cardiomyopathy, non-compaction, restrictive cardiomyopathy/ constrictive pericarditis, heart failure, diastolic dysfunction, heart transplant, implanted pacemaker, coronary artery disease and cardiovascular disease risk factors. Evidence showing changes in LV rotation due to various types of heart disease was supported by evidence with limited to moderate methodological quality.

**Conclusions:**

Despite a relatively low quality and volume of evidence, the literature consistently shows that heart disease leads to marked changes in LV rotation, while rotational systolic-diastolic coupling is preserved. No prognostic information exists on the potential value of rotational measures of LV function. The literature suggests that measures of LV rotation may aid in diagnosing subclinical aortic stenosis and diastolic dysfunction.

## Background

In recent years, there has been increased interest in quantifying left ventricular (LV) rotation.[1-6] Using basal and apical views of the heart (and occasionally mid-ventricular) the myocardium can be digitally “tagged” (using magnetic resonance imaging (MRI)) or “tracked” (echocardiography), using specialized software, and its motion analyzed. Although a variety of nomenclature exists, most commonly in the literature, LV twist is estimated by calculating the maximal instantaneous difference in rotation between the apical and basal levels in the short axis plane. Torsion is calculated by dividing twist by the longitudinal length between the two recorded short-axis levels (ie. LV length). Left ventricular untwist refers to the amount of twist that occurs during diastole [7,8]. Echocardiography with tissue tracking and MRI with tissue tagging are the most commonly performed techniques for evaluating these parameters [9-11]. The use of MRI for evaluating rotation of the LV is the considered the most accurate technique and has been shown to correlate well with the tissue Doppler technique[11] and speckle tracking echocardiography (STE) [9,12]. It should be noted that a trade-off exists between MRI and STE for the measurement of LV rotation. Although it is considered more accurate, MRI requires considerably more time for acquisition, has less temporal resolution, and is less affordable and accessible in most research and clinical environments. Indeed, the recent surge in the number of publications evaluating LV rotation has coincided with the arrival of ultrasound tissue tracking [13].

In young healthy individuals, systole of the LV is associated with counterclockwise rotation of the apical level while the basal level rotates clockwise (when viewed from the apex). Rotational motion, according to mathematical models, depends disproportionately on fibers arranged in a helical manner around the LV [14]. It has been suggested that up to 40% of stroke volume is produced from twisting forces within the LV [6]. Additionally, myocardial energy efficiency is thought to be dependent on LV twist; both by normalizing the fiber shortening of the endocardial and epicardial layers during contraction, and by creating sufficient transmitral pressure to aid in ventricular filling during diastole. Stored energy, in the spring like cardiac protein titin, is considered to be largely influenced by twist [15]. The resulting early untwisting (which primarily occurs before the opening of the mitral valve) beneficially influences early diastolic filling [16]. Left ventricular rotation parameters have been shown to have weak but significant negative correlations to afterload and male gender while possessing a weak positive relationship with heart rate [17,18]. Left ventricle rotation is also affected in various heart diseases including but not exclusively: aortic stenosis [19], heart failure [20], cardiomyopathy [21], heart transplant [5], diastolic dysfunction [3], and myocardial infarction (MI) [22]. The purpose of this systematic review is to present a synopsis of the scientific literature investigating the impact of various heart diseases on LV rotation. We feel a review characterizing heart disease related changes in ventricular rotation is necessary to summarize the current trends, as the interest in these measures increases. Our objective is to review systematically the evidence measuring LV rotational changes in various heart diseases compared to healthy controls.

## Methods

**Figure 1:**
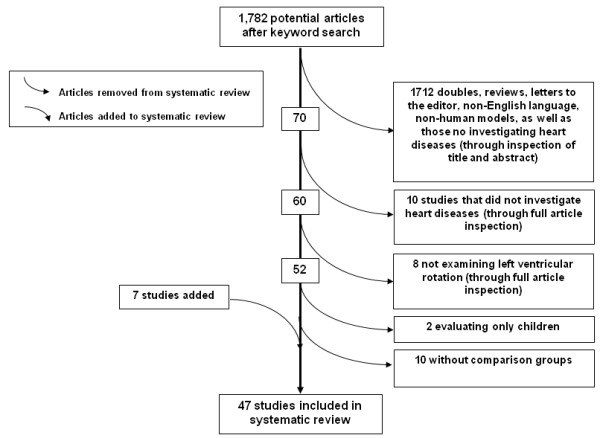
Article decisions - flow of studies through the review.

A keyword literature search for all scientific publications from 1950 to present investigating the interaction between heart disease and LV rotational parameters was conducted using the following online databases: MEDLINE, EMBASE, Cochrane Library, ACP Journal Club, DARE, CCTR, CMR, HTA, NHSEED, PsycINFO, SPORTDiscus and CINAHL. Heart disease key words – *heart disease, cardiovascular disease, heart failure, heart transplant, valve stenosis, aortic stenosis, cardiomyopathy, myocardial infarction, transplant* – and rotational function keywords – *rotation, twisting, untwisting, recoil, twist, torsion, torsional* – as well as ventricular anatomy phrases – *ventricular, ventricle, heart, cardiac, and myocardial* - were paired by permutation (Table 1). A total of 1,782 papers were found after which duplicates, review papers, letters to the editor, those not in English, those without comparable groups, those examining extremely rare heart diseases, and those not evaluating LV rotational outcomes in human adults were removed from the sample; leaving a total of 40 articles. Seven additional papers [2,5,22-25] were added to the sample as a result of cross referencing, leaving a total of 47 articles (Figure 1).

**Table 1:** Results of the OVID (MEDLINE, EMBASE, ACP, Cochrane Library, DARE, CCTR, CMP, HTA, NHSEED) literature search

**#**	**Searches (18 December 2010)**	**Results**
*Region of Interest Search Term*		
1	Ventricular	493973
2	Ventricle	341745
3	Heart	2225277
4	Cardiac	871838
5	myocardial	567273
*Rotation Characteristic Search Term*		
6	Rotation	114762
7	Twisting	4910
8	Untwisting	580
9	Recoil	4625
10	Twist	9589
11	Elastic recoil	2019
12	Torsion	29064
13	Torsional	9246
*Heart Disease Search Term*		
14	Heart disease	334979
15	Cardiovascular disease	205473
16	Heart failure	275006
17	Heart transplant	12545
18	Valve stenosis	59694
19	Aortic stenosis	17785
20	Cardiomyopathy	109271
21	Myocardial infarction	298842
22	Transplant	197267
*Combined Region of Interest Search Terms*		
23	1 or 2 or 3 or 4 or 5	2694602
*Combined* Rotation Characteristic Search Term		
24	6 or 7 or 8 or 9 or 10 or 11 or 12 or 13	164444
	*Combined* Heart Disease Search Term		
25	14 or 15 or 16 or 17 or 18 or 19 or 20 or 21 or 22 or 23	1937593	
*Combined Population of Interest, Outcome Variable, and Intervention Strategy Search Terms*			
26	23 and 24 and 25	1634	

Please note this does not include the results of the EBSCO (PsychINFO, SPORTDiscuss and CINAHL) literature search.

An evaluation of the methodological quality of each article was completed by two reviewers (AP, AC) and confirmed by a third reviewer with expertise in systematic reviews (DW) using the Downs and Black (D&B) tool for non-RCTs [26]. The highest and therefore most methodologically strong score attainable for a given research article is 27 for the D&B Tool. Higher points indicate a superior methodological quality. Further, the level of evidence was evaluated using a five level scale [26] (simplified form of Sackett) [27] where Level 1 (the highest level of evidence) = RCT with a high methodological quality score; Level 2 = a RCT with a low methodological quality score, a non-randomized prospective-controlled study, or a cohort study; Level 3 = a case–control study; Level 4 = a pre- and post-test or a case series; and Level 5 (the lowest level of evidence) = an observational report or case report with only a single subject [28]. Librarians from the University of British Columbia and all authors approved this systematic process. Ranking scores were performed in duplicate after which any discrepancies were solved by discussion.

Operational definitions were developed to streamline comparisons of primary outcome variables between studies. Rotation was defined as the rotary motion (in degrees) of either the apex, base, or mid-ventricle. Also, as viewed from the apex of the heart, counterclockwise rotation is denoted by a positive angle (degrees) while clockwise rotation is marked by a negative angle (degrees).[29,30] Twist was further defined as the maximal instantaneous basal to apical angle difference (degrees).[31-33] Torsion was then defined as twist divided by end-diastolic LV length (between the two short axis images) (degrees/cm).[7,29,30,34] Mean values for systolic peak rotation, systolic basal rotation, peak systolic twist, twist rate, peak torsion, diastolic peak untwist, untwist rate, and time to peak untwist were recorded from each article which provided data.

Where two or more articles reported mean differences between a given cardiovascular disorder, the average and standard deviation of the percent difference was calculated. These values are presented graphically in a series of figures throughout this article. Percent differences between groups are reported as the difference relative to healthy controls in *absolute amplitude*. For example, a percent reduction in both a negative rotation at the base and a positive rotation at the apex are both reported as negative values.

## Results

The articles selected were categorized into thirteen groups according to heart disease. Within the text we have provided a summary table where the overall findings from the moderate to strong articles within each heart disease may be found (Table 2). In order to have a general finding presented in the summary table, two or more articles of moderate to strong methodological quality using the same imaging technique must have reported on a given LV rotation parameter. In the online supplement we have provided comprehensive tables for each heart disease group which describes specific details and rankings of each article in order of descending methodological quality.

**Table 2:** Summary of difference in left ventricular rotation between heart disease patients and healthy controls

**Heart Disease**	**D&B Quality Score Mean (SD)**	**No. Articles**	**Sample Size** **Mean (SD)**	**Apical Rotation (°)**	**Basal Rotation (°)**	**Twist (°)**	**Twist Rate (°/s)**	**Torsion (°/length)**	**Untwist Rate (°/s)**	**Time to Peak Untwist**	**Reverse Rotation**
Aortic Stenosis	STE = 17 (2) MRI = 16 (1)	4 2	N = 162 N = 25	**↑ ↑**	**↔**	**↑ ↑**	**↔/↑**	**↑**		**↔/↑ ↑**	
Myocardial Infarction	STE = 19 (3) MRI = 15 (1)	3 3	N = 129 N = 73	**↓ ↔/↓**	**↓ ↔**	**↓**				**↑**	
Hypertrophic Cardiomyopathy	STE = 18 (3) MRI = 12 (1)	7 2	N = 257 N = 15	**↔ ↔/↓**	**↑ ↔**	**↓/↔/↑**			**↔**	**↔/↑**	
Dilated Cardiomyopathy	STE = 19 (1)	6	N = 294	**↓**	**↓**	**↓**		**↓**	**↓**	**↑**	Yes
Non-compaction	STE = 18 (1)	2	N = 30	**↓**	**↔**	**↓**	**↓**	**↓**			Yes
Heart Failure	STE = 21 (3.5)	3	N = 149	**↓**	**↓**	**↓**					Yes
Diastolic Dysfunction	STE = 20 (2)	6	N = 347	**↔**	**↔/↑**	**↔**	**↑**			**↔**	

N; total number of subjects in heart disease group from all publications in group, D & B; Downs and Black score, MRI; magnetic resonance imaging, 2D-STE; two dimensional speckle tracking echocardiography, **↑**; significant increase in heart disease group as compared to healthy controls, **↓** significant decrease in heart disease group as compared to healthy controls, **↔**. Note that for HCM, we did not report the two articles which used VVI as we required two or more articles for any value reported in this table and the two articles using VVI reported the same results in both publications.

Heart disease groups include: 1-*Aortic Stenosis* (n = 7, Online Table 3), 2-*MI* (n = 6, Online Table 4), 3-*Hypertrophic Cardiomyopathy* (HCM; n = 11, Online Table 5), 4-*Dilated Cardiomyopathy* (n = 9, Online Table 6), 5-*Non-Compaction* (n = 2, Online Table 7), 6-*Systolic Heart Failure* (SHF; n = 5, Online Table 8), 7-*Heart Failure Normal Ejection Fraction/Diastolic Dysfunction (HFnEF)* (n = 6, Online Table 9), 8-*Heart Transplant* (n = 1, Online Table 7), 9-*Implanted Pacemaker* (n = 1, Online Table 7), 10-*Cardiovascular Disease Risk Factors* (n = 1, Online Table 7), 11-*Restrictive Cardiomyopathy/Constrictive Pericarditis,* (n = 1, Online Table 7) and 12-*Coronary Artery Disease* (n = 1, Online Table 7). Groups 8–12 were discussed in combination as each consisted of only one published article. Note that one paper examined both hypertrophic and dilated cardiomyopathy [35], another both non-compaction and dilated cardiomyopathy [36], another heart failure and diastolic dysfunction [20], while an additional paper investigated both diastolic dysfunction and cardiovascular disease risk factors with no overt disease [37]. All articles, except one prospective control trial in the HCM section [25] were of the case control design (level 3 evidence). The D&B Tool scores ranged from 9–23 out of 27 (limited to strong methodological strength) [38-40]. Standardizing the location where short axis basal and apical images are located is an important consideration. Although this is not commonly a concern with MRI, as image location can be chosen very accurately, ultrasound collection of the apical location in particular is a challenging task. Only one article reviewed here reported collecting the superior short axis image in a non-traditional location (papillary muscle level) [41]. Further to this, three others simply did not describe their locations (although they were referred to as basal and apical) [20,42,43]. In light of this, we feel the short axis ultrasound image locations were well standardized within the literature. Finally, although a small number of articles do not explicitly state the position of the participants during collection, the vast majority report image acquisition occurring while the participant is resting in the supine or lateral decubitus position.

**Table 3:** Difference in left ventricular rotational parameters between participants with aortic stenosis as compared to healthy controls

**Study Details**	**Matched Controls**	**Apical Rotation**	**Basal Rotation**	**Twist**	**Torsion**	**Twist Rate**	**Peak Untwist Rate**	**Time to Peak Untwist**	**Additional Notes**
Popescu et al. 2010 Case–control Level 3 D&B = 19 2D-STE**	**No**	**↑**	**↔**	**↑**	**↑**	**↑**	**↔**	**↔**	Time to peak apical untwisting rate was longer in those with aortic stenosis.
Tzemos et al. 2008 Case–control Level 3 D&B = 19 2D-VVI *	**Yes**			**↑**					Sample comprised of women.
Stuber et al. 1999 Case–control Level 3 D&B = 17 MRI Tagging	**No**	**↑**		**↑**	**↑**			**↑**	Time to peak apical untwisting velocity was increased in those with aortic stenosis.
Van Dalen et al. 2011 Case–control Level 3 D&B = 16 2D-STE***	**Yes**	**↑**	**↔**	**↑**		**↔**	**↑**	**↑**	
Carasso et al. 2009 Case–control Level 3 D&B = 15 2D-STE*	**Yes**			**↓**					Aortic stenosis compared to healthy controls.
Nagel et al. 2000 Case–control Level 3 D&B = 15 MRI Tagging	**No**	**↑**	**↓**	**↑**			**↓**	**↑**	
Sandstede et al. 2002 Case–control Level 3 D&B = 13 MRI Tagging	**Yes**	**↑**	**↔**	**↑**					

D & B; Downs and Black score, MRI; magnetic resonance imaging, 2D-STE; two dimensional speckle tracking echocardiography, 2D-VVI; two dimensional velocity vector imaging, ***indicates that mitral leaflets and luminal obliteration were used for identifying the basal and apical images respectively, **indicates that mitral leaflets and location inferior to papillary muscle were used for identifying the basal and apical images respectively, * indicates that land marking for short axis images was poorly described, **↑**; significant increase in heart disease group as compared to healthy controls, **↓** significant decrease in heart disease group as compared to healthy controls, **↔**; no significant difference between heart disease group as compared to healthy controls.

**Table 4:** Difference in left ventricular rotational parameters between participants with prior myocardial infarction as compared to healthy controls

**Study Details**	**Matched Controls**	**Apical Rotation**	**Basal Rotation**	**Twist**	**Torsion**	**Twist Rate**	**Time to Peak Twist**	**Time to Peak Untwist**	**Additional Notes**
Govind et al. 2010 Case–control Level 3 D&B = 23 2D-STE***	**Yes**	**↓**	**↓**	**↓**					Participants with MI and normal EF (>40%) had elevated twist and apical rotation rate as compared to participants with MI and low EF (<40%).
Bansal et al. 2008 Case–control Level 3 D&B = 17 2D-STE**	**No**	**↓**	**↓**		**↓**				Increasing number of infarcts was related to decreased basal rotation and torsion but not apical rotation.
Takeuchi et al. 2007 Case–control Level 3 D&B = 17 2D-STE**	**Yes**	**↔/↓**	**↔**	**↔/↓**		**↔/↓**	**↔**	**↔/↑**	Anterior MI participants were divided into those with normal (≥45%) and reduced (<45%) EF. Only reduced EF participants had reduced twist and apical rotation as well as time to peak untwist as compared to controls.
Nagel et al. 2000 Case–control Level 3 D&B = 15 MRI Tagging	**No**	**↓**	**↔**				**↔**	**↑**	Apical rotation is reduced in those with anterolateral MI but no difference between groups in mid level or basal rotation occurred. In a subgroup of anterolateral MI participants with accompanying LV aneurism, there is a complete loss of apical rotation and a reversal of mid level rotation that rotates with the base instead of the apex as in healthy controls.
Garot et al. 2002 Case–control Level 3 D&B = 15 MRI Tagging	**No**								Only mid-ventricle (between apex and base) rotation was reported. Mid-ventricle rotation was reduced in those with MI. Those with normal EF (>48%) had higher rotation than those with low EF (<48%).
Setser et al. 2007 Case–control Level 3 D&B = 14 MRI Tagging	**No**	**↔**	**↔**	**↓**					Ischemic cardiomyopathy/MI compared to healthy controls.

D & B; Downs and Black score, MRI; magnetic resonance imaging, 2D-STE; two dimensional speckle tracking echocardiography, *** indicates that mitral leaflets and luminal obliteration were used for identifying the basal and apical images respectively, ** indicates that mitral leaflets and location inferior to papillary muscle were used for identifying the basal and apical images respectively, LV; left ventricle, EF; ejection fraction, **↑**; significant increase in heart disease group as compared to healthy controls, **↓** significant decrease in heart disease group as compared to healthy controls, **↔**; no significant difference between heart disease group as compared to healthy controls.

**Table 5:** Difference in left ventricular rotational parameters between participants with hypertrophic cardiomyopathy compared to healthy controls

**Study Details**	**Matched Controls**	**Apical Rot**	**Basal Rot**	**Twist**	**Time to Peak Twist**	**Peak Untwist Rate**	**Untwist Rate**	**Time to Peak Untwist**	**Early Untwist Rate**	**Additional Notes**
Chang et al. 2010 Case–control Level 3 D&B = 23 2D-STE***	**Yes**	**↓**	**↔**	**↓**	**↔**	**↓**		**↔**	**↓**	Peak untwist rate P-value = 0.07
Buakhamsri et al. 2009 Case–control Level 3 D&B = 19 2D-STE***	**Yes**						**↔**			
van Dalen et al. 2009 Case–control Level 3 D&B = 18 2D-STE***	**Yes**	**↔**	**↑**	**↑**	**↔**				**↓**	HCM were separated into groups with either sigmoidal or reverse septal morphology. Both sub-groups had increased basal rotation. Only the sigmoidal group had increased apical rotation and twist.
Carasso et al. 2010 Case–control Level 3 D&B = 18 2D-VVI**	**Yes**			**↔**						Twist time was reduced and untwist time increased in those with HCM.
Carasso et al. 2008 Case–control Level 3 D&B = 18 2D-VVI**	**Yes**	**↔**	**↔**	**↔**						Mid-level rotation was in the opposite direction as compared to healthy controls (clockwise instead of counter clockwise).
Wang et al. 2009 Case–control Level 3 D&B = 17 2D-STE***	**Yes**			**↔**			**↔**			Time to untwisting was lowest in controls, elevated in HCM group and increased again in hypertrophic obstructive cardiomyopathy group.
Notomi et al. 2006 PCT Level 2 D&B = 17 2D-STE***	**Yes**			**↔**		**↔**				At rest, no significant differences were reported. During exercise, peak untwisting velocity and peak systolic twist increased in control group but not in HCM group.
van Dalen et al. 2009 Case–control Level 3 D&B = 17 2D-STE***	**Yes**	**↔**	**↑**	**↑**		**↓**		**↑**	**↓**	Similar to above, HCM were separated into groups with either sigmoidal or reverse septal morphology. Both sub-groups had increased basal rotation. Only the sigmoidal group had increased apical rotation and twist.
Abozguia et al. 2010 Case–control Level 3 D&B = 14 2D-STE**	**Yes**	**↔**	**↔**	**↔**						In those with non-obstructive HCM there was a delay in reaching 25% untwist.
Maier et al. 1992 Case–control Level 3 D&B = 13 MRI Tagging	**N/A**	**↓**	**↔**							
Young et al. 1994 Case–control Level 3 D&B = 10 MRI Tagging	**N/A**	**↔**	**↔**	**↑**						Twist was calculated by subtracting the base rotation from apical rotation.

D & B; Downs and Black score, MRI; magnetic resonance imaging, 2D-STE; two dimensional speckle tracking echocardiography, 2D-VVI; two dimensional velocity vector imaging, *** indicates that mitral leaflets and luminal obliteration were used for identifying the basal and apical images respectively, ** indicates that mitral leaflets and location inferior to papillary muscle were used for identifying the basal and apical images respectively, Apical Rot; apical rotation, Basal Rot; basal rotation, HCM; hypertrophic cardiomyopathy. **↑**; significant increase in heart disease group as compared to healthy controls, **↓** significant decrease in heart disease group as compared to healthy controls, **↔**; no significant difference between heart disease group as compared to healthy controls.

**Table 6:** Difference in left ventricular rotational parameters between participants with dilated cardiomyopathy as compared to healthy controls

**Study Details**	**Matched Controls**	**Apical Rot**	**Basal Rot**	**Twist**	**Torsion**	**Time to Peak Twist**	**Peak Untwist Rate**	**Untwist Rate**	**Time to Peak Untwist**	**Additional Notes**
Buakhamsri et al. 2009 Case–control Level 3 D&B = 19 2D-STE***	**No**							**↓**		DCM separated into sub-groups who had either a wide or narrow QRS complex. Narrow-QRS group had reduced untwisting velocity as compared to controls while wide-QRS complex had a further reduced untwisting velocity.
van Dalen et al. 2010 Case–control Level 3 D&B = 19 2D-STE***	**Yes**	**↓**	**↔**	**↓**			**↓**	**↓**	**↑**	
Liu et al. 2010 Case–control Level 3 D&B = 19 2D-VVI***	**Yes**	**↓**	**↓**	**↓**	**↓**			**↓**		
Popescu et al. 2009 Case–control Level 3 D&B = 19 2D-STE**	**No**	**↓**	**↓**		**↓**					DCM were separated into sub-groups who had normal or reversed apical rotation. Torsion was lower in those with normal rotation compared to controls. Torsion was further reduced in those with opposite apical rotation (essentially torsion was lost). Basal rotation was not different between the two DCM groups. 31/50 participants had opposite rotation at either the base or the apex.
Meluzin et al. 2009 Case–control Level 3 D&B = 18 2D-STE***	**Yes**	**↓**	**↓**	**↓**	**↓**					18/37 participants had opposite rotation of either the apex or base.
Saito et al. 2009 Case–control Level 3 D&B = 18 2D-STE**	**Yes**	**↓**	**↓**	**↓**			**↓**		**↑**	
Kanzaki et al. 2006 Case–control Level 3 D&B = 17 MRI Tagging	**No**	**↓**	**↓**	**↓**	**↓**	**↓**				In those with DCM, the apex turned with the base at mid-systole and did not rotate counter clockwise throughout contraction as in healthy controls.
van Dalen et al. 2008 Case–control Level 3 D&B = 17 2D-STE***	**Yes**	**↓**	**↔**	**↓**		**↔**				
Sade et al. 2008 Case–control Level 3 D&B = 15 2D-STE**	**No**	**↓**	**↓**	**↓**	**↓**	**↑**				DCM separated into sub-groups who had either ischemic or non-ischemic disease. Both groups had similar LV rotation characteristics. 15/34 participants had opposite rotation at either the base or the apex.

D & B; Downs and Black score, MRI; magnetic resonance imaging, 2D-STE; two dimensional speckle tracking echocardiography, 2D-VVI; two dimensional velocity vector imaging, ***indicates that mitral leaflets and luminal obliteration were used for identifying the basal and apical images respectively, **indicates that mitral leaflets and location inferior to papillary muscle were used for identifying the basal and apical images respectively, Apical Rot; apical rotation, Basal Rot; basal rotation, DCM; dilated cardiomyopathy, **↑**; significant increase in heart disease group as compared to healthy controls, **↓** significant decrease in heart disease group as compared to healthy controls, **↔**; no significant difference between heart disease group as compared to healthy controls.

**Table 7:** Difference in left ventricular rotational parameters between participants with various cardiovascular risk factors, right ventricle apical pacing, non-compaction, transplants, coronary artery disease, restrictive cardiomyopathy and constrictive pericarditis as compared to healthy controls

**Study Details**	**Matched Controls**	**Apical Rot**	**Basal Rot**	**Twist**	**Twist Rate**	**Time to Peak Twist**	**Peak Untwist Rate**	**Untwist Rate**	**Additional Notes**
Sengupta et al. 2008 Case–control Level 3 D&B = 20 2D-STE***	**Yes**	**↔**	**↔**	**↔**					Restrictive cardiomyopathy did not have significantly different torsion compared to healthy controls.
Sengupta et al. 2008 Case–control Level 3 D&B = 20 2D-STE***	**Yes**	**↓**	**↔**	**↓**					Apical rotation rate, twist and torsion was reduced in those with constrictive pericarditis.
Paetsch et al. 2005 Case–control Level 3 D&B = 19 MRI Tagging	**Yes**	**↓**							Measures were not taken at rest. Measures were collected during low or high doses of dobutamine. At both doses, those with coronary heart disease had reduced measures as compared to controls. Increased time to untwist was reported in clinical population.
Delgado et al. 2009. PCT Level 2 D&B = 18 2D-STE***	**Yes**	**↓**	**↓**	**↓**					Right ventricle apical pacing compared to healthy controls.
Mizuguchi et al. 2008 Case–control Level 3 D&B = 17 2D-STE**	**Yes**			**↔**	**↔**			**↔**	Various cardiovascular risk factors compared to healthy controls.
van Dalen et al. 2008 Case–control Level 3 D&B = 17 2D-STE***	**Yes**	**↓**	**↔/↑**	**↓**	**↓**	**↔**			In all non-compaction participants, the base and apex rotated in the same direction. Those with clockwise rotation had opposite (reduced) apical rotation but normal basal rotation. Those with counter clockwise rotation had reduced apical rotation and opposite (increased) basal rotation.
Bellavia et al. 2010 Case–control Level 3 D&B = 19 2D-STE***	**Yes**	**↓**	**↔/↓**	**↓**	**↓**				Non-compaction with normal EF (≥50%) was not different in basal rotation from healthy controls whereas those with low EF (<50%) had reduced basal rotation.
Esch et al. 2009 Case–control Level 3 D&B = 14 2D-STE**	**Yes**			**↔**			**↔**		Heart transplants regress to recipient matched rotation characteristics (instead of maintaining donor age matched rotation). Compared to recipient matched controls, heart transplants had reduced untwisting response to exercise. Both recipient age matched controls and transplant recipients had reductions in twist with exercise whereas donor matched had increased twist with exercise.

D & B; Downs and Black score, MRI; magnetic resonance imaging, 2D-STE; two dimensional speckle tracking echocardiography, EF; ejection fraction, ***indicates that mitral leaflets and luminal obliteration were used for identifying the basal and apical images respectively, **indicates that mitral leaflets and location inferior to papillary muscle were used for identifying the basal and apical images respectively **↑**; significant increase in heart disease group as compared to healthy controls, **↓** significant decrease in heart disease group as compared to healthy controls, **↔**; no significant difference between heart disease group as compared to healthy controls.

**Table 8:** Difference in left ventricular rotational parameters between participants with heart failure as compared to healthy controls

**Study Details**	**Matched Controls**	**Apical Rotation**	**Basal Rotation**	**Twist**	**Additional Notes**
Zhang et al. 2008 Case–control Level 3 D&B = 23 2D-STE*	**Yes**	**↓**	**↓**	**↓**	
Bertini et al. 2009 Case–control Level 3 D&B = 23 2D-STE**	**Yes**	**↓**	**↓**	**↓**	
Fuchs et al. 2004 Case–control Level 3 D&B = 23 MRI Tagging	**No**	**↓**	**↓**		Diastolic basal rotation was similar between controls and those with heart failure. Diastolic apical rotation was reduced in those with heart failure.
Russel et al. 2009 Case–control Level 3 D&B = 18 MRI Tagging	**No**			**↓**	20/34 participants with heart failure had reversed rotation patterns whereas no healthy controls did.
Wang et al. 2008 Case–control Level 3 D&B = 17 2D-STE*	**No**			**↓**	

D & B; Downs and Black score, MRI; magnetic resonance imaging, 2D-STE; two dimensional speckle tracking echocardiography, **indicates that mitral leaflets and location inferior to papillary muscle were used for identifying the basal and apical images respectively, * indicates that land marking for short axis images was not a widely accepted technique or poorly described, **↑**; significant increase in heart disease group as compared to healthy controls, **↓** significant decrease in heart disease group as compared to healthy controls, **↔**; no significant difference between heart disease group as compared to healthy controls.

**Table 9:** Difference in left ventricular rotational parameters between participants with diastolic dysfunction as compared to healthy controls

**Study Details**	**Matched Controls**	**Apical Rotation**	**Basal Rotation**	**Twist**	**Torsion**	**Twist Rate**	**Time to Peak Twist**	**Peak Untwist Rate**	**Untwist Rate**	**Time to Peak Untwist**	**Additional Notes**
Wang et al. 2008 Case–control Level 3 D&B = 23 2D-STE*	**No**			**↔**							Diastolic dysfunction group had normal ejection fraction but had diastolic heart failure.
Perry et al. 2008 Case–control Level 3 D&B = 20 2D-STE**	**No**										Early diastolic apical untwist was reduced in abnormal relaxation vs. controls, was further reduced in pseudonormal relaxation and reduced additionally in restrictive filling.
Phan et al. 2009 Case–control Level 3 D&B = 20 2D-STE**	**Yes**	**↔**	**↔**		**↔**		**↔**			**↔**	Diastolic dysfunction group had normal ejection fraction but had diastolic heart failure.
Jang et al. 2009 Case–control Level 3 D&B = 20 2D-STE***	**No**	**↔**		**↔**							Participants with diastolic dysfunction were separated into those with intermediate (11.1) or elevated (18.2) E/E' ratio. Apical rotation was borderline increased in those with intermediate but not elevated E/E' (P = 0.07).
Park et al. 2008 Case–control Level 3 D&B = 19 2D-STE***	**Yes**	**↑**	**↑**	**↑**		**↑**	**↔**	**↑**	**↑/↓**	**↔**	Grade 1 diastolic dysfunction reported in table. Grade 2 group was not different from controls in any parameter. Grade 3 participants had reduced untwisting rate as compared to controls.
Mizuguchi et al. 2008 Case–control Level 3 D&B = 17 2D-STE**	**Yes**			**↔**		**↑**					Diastolic dysfunction group had reduced E/A (< 1) ratio but preserved ejection fraction.

D & B; Downs and Black score, MRI; magnetic resonance imaging, 2D-STE; two dimensional speckle tracking echocardiography, ***indicates that mitral leaflets and luminal obliteration were used for identifying the basal and apical images respectively, **indicates that mitral leaflets and location inferior to papillary muscle were used for identifying the basal and apical images respectively, * indicates that land marking for short axis images was not a widely accepted technique or poorly described, E/A ratio; ratio of peak velocity of early filling to peak velocity of late filling, E/E’ ratio; ratio of mitral peak velocity of early filling to early diastolic mitral annular velocity, **↑**; significant increase in heart disease group as compared to healthy controls, **↓** significant decrease in heart disease group as compared to healthy controls, **↔**; no significant difference between heart disease group as compared to healthy controls.

### Aortic stenosis

#### *Systolic parameters*

Of the seven moderate strength papers (D&B scores ranged from 15–19) to report on LV rotation in those with aortic stenosis, six papers showed agreement that LV twist is elevated (Online Figure 2). The lone paper that reported reduced LV twist in those with aortic stenosis was removed from this analysis as the authors chose to use a modified technique examining twist relative to the mid-ventricular instead of the basal level [41]. Left ventricular torsion was investigated in two of the strongest quality articles; both showing an elevation as compared to healthy controls (Online Figure 2) [8,44]. Also, five moderate quality papers reported on the maximal apical rotation in those with aortic stenosis, all showing increased apical rotation as compared to healthy controls [8,44-47]. Of the four papers that reported individual basal rotation, three reported no change in basal rotation [8,45,47] while one of the lower quality articles showed a reduction [46].

**Figure 2:**
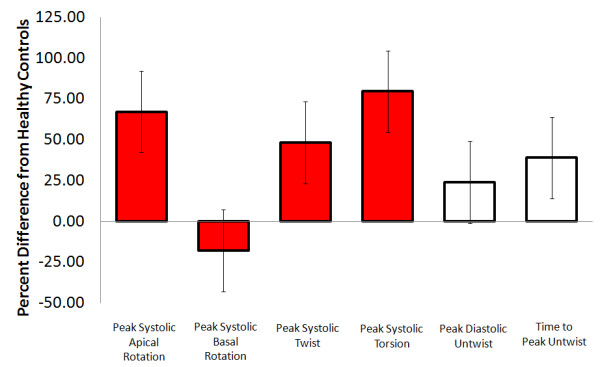
**Aortic stenosis -** Average percent difference in left ventricular peak systolic apical rotation (Range: Control; 6.8 to 5.7, Patients; 12 to 22.2 degrees),[8,44-46] peak systolic basal rotation (Range: Control; -4.2 to −6.2, Patients; -2.4 to −6.7 degrees),[8,45,46] peak systolic twist (Range: Control; 8 to 20.8, Patients;12 to 22.2 degrees), [8,45,46] peak systolic torsion (Range: Control; 0.6 to 2.7, Patients;1.4 to 3.4 degrees/cm),[8,44] peak diastolic untwist (Range: Control; -54.8 to −143, Patients;-80 to −158 degrees/sec) [8,44] and time to peak untwist (Range: Control; 56 to 115, Patients; 103 to 115 ms) [8,46] between those with aortic stenosis and healthy controls as reported in existing articles. Systolic parameters denoted by red filled boxes. Diastolic parameters denoted by empty boxes.

### *Diastolic parameters*

There was significant disagreement in the literature regarding peak untwist rate in those with aortic stenosis, as increases [47], decreases [46] and no differences were reported (Online Figure 2) [8]. This disagreement is likely related to poorly matched controls, as other than the article from van Dalen, which showed an increase in peak untwist rate, the two remaining articles in this group had control participants that were on average 20 and 30 years younger than patients [8,46]. Taken together, these results suggest that peak untwist rate may be increased in comparison to age matched controls, however the age related reduction in LV untwist may confound this finding in studies with much younger controls [48]. Finally, three articles reported a prolonged time to peak untwist in those with aortic stenosis [44,46,47], while one article showed no change [8]. The latter article reported that time to peak apical untwist was prolonged but not time to peak basal untwist however, suggesting some sort of disruption in temporal parameters of diastolic rotation.

#### Conclusions

The literature shows, from the available moderate strength evidence, that aortic stenosis (LV pressure overload) is associated with an average 75% increase in systolic apical rotation but very little change (perhaps a small decrease) in basal rotation. It is less clear how diastolic rotation is related to aortic stenosis. It is interesting however that time to peak diastolic untwist was prolonged in aortic stenosis. This may be due to a greater time requirement for peak passive force generation from compressed cardiac spring proteins; owing to greater compression during systole.

### Myocardial infarction

Six published articles investigated LV rotation in those with MI as compared to healthy controls [22,24,49-52]. Downs and Black scores ranged from 14–23 (moderate to strong methodological quality).

### *Systolic parameters*

Five papers reported a reduction in LV twist [24,49,51,52] or torsion [50] in those with prior MI. Also, four articles reported a decrease in apical rotation in those with MI, although one article by Takeuchi et al. showed no difference [51]. The discrepancy is likely explained more by inclusion criteria than methodological quality as approximately 50% of MI participants had relatively high ejection fractions (EF) (>45%) in Takeuchi’s work. When the prior MI group was divided into those with high and low EF, a significant reduction was found for twist and apical rotation in the *low EF* sub-group only [51]. This relationship between twist and low EF in those with prior MI was confirmed in work by and Govind and colleagues [49]. Similarly, two of the five articles reporting on basal rotation showed a reduction in MI [49,50], whereas three showed no difference between groups (Online Figure 3) [22,51,52]. Although the two articles reporting decreases in basal rotation were of higher methodological quality than the three showing no difference, we feel the discrepancy can be best explained by work by Bansal et al., which showed region of infarct can influence greatly LV rotational dysfunction [50]. For example, the three articles to report no different in systolic basal rotation examined only those with anterior infarction, while the other two articles consisted of a more heterogeneous sample with several regions of infarct.

**Figure 3:**
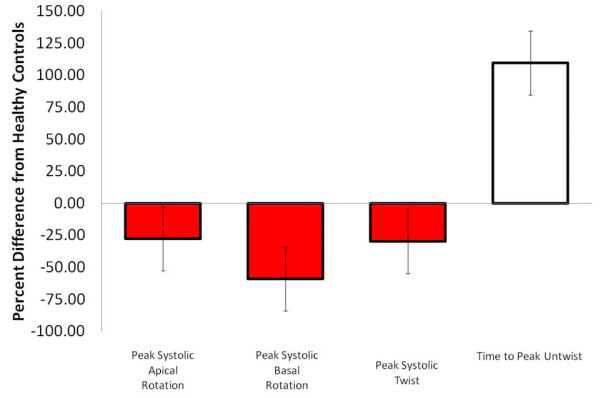
**Myocardial infarction -** Average percent difference in left ventricular peak systolic apical rotation (Range: Control; 5.2 to 12.5, Patients; 4.1 to 8.8 degrees),[22,49-52] peak systolic basal rotation (Range: Control; -3.1 to −8.8, Patients; -1 to −5.9 degrees),[22,49-52] peak systolic twist (Range: Control; 9.3 to 21.8, Patients;7.7 to 13.3 degrees),[24,49,51] and time to peak untwist (Patients; 97 to 122 ms delayed) [22,51] between those with myocardial infarction and healthy controls as reported in existing articles. Systolic parameters denoted by red filled boxes. Diastolic parameters denoted by empty boxes.

### *Diastolic parameters*

Three moderate strength articles which reported on LV rotation in diastole suggest that both untwisting rate and timing are negatively affected by MI. Specifically, two articles showed an increased time to peak untwisting velocity in those with prior MI (Online Figure 3) [22,51], while one article showed that early untwist rate is reduced [52].

#### Conclusions

There is moderate to strong level three evidence (somewhat reliable) that rotation characteristics in both systole and diastole are altered in those with prior MI. Specifically, there is agreement in the literature that twist and apical rotation are reduced in MI, however *this relationship occurs only when EF is affected* by infarction. Also, there is moderately strong evidence that time to peak untwist is longer in those with MI and early untwist rate is reduced; likely the result of systolic-diastolic coupling. Clearly, more work is needed, especially examining LV diastolic rotational parameters in those with MI. Work from Bansal and colleagues highlights a very interesting issue within studies of MI, whereby perhaps global markers of twist and rotation are not suitable for this population unless evaluating differences according to region of infarct [50].

### Hypertrophic cardiomyopathy

Eleven published articles investigated LV rotation in those with HCM as compared to healthy controls [21,23,25,31-33,35,53-56]. One article from this group was a prospective controlled trial (level two evidence) and not a case–control study [25]. Down and Black scores ranged from 10–23 (limited to strong methodological quality).

### *Systolic parameters*

Those with HCM were widely reported to have no difference in apical rotation [21,23,31,32,56] however two articles showed a significant reduction.[53,55] As the methodological strength was similar for all seven articles, we feel the discrepancies are better explained by methodological differences. The two latter papers were comprised of one article that investigated only those with apical HCM [55] and another which did not report statistics for the difference claimed within the abstract and discussion [53]. In contrast, the five articles which reported no difference in apical rotation were comprised of a relatively heterogeneous group of HCM patients, with accompanying statistical procedures.

A total of seven articles reported on basal rotation in HCM, however two did not report statistics and/or had a small sample size (n = 7 [23], n = 8 [53]) while a third investigated only those with apical HCM [55]. Following this, only four were methodologically comparable and valid. These four articles (which had moderate methodological strength, large sample sizes, comparable groups and used STE) consisted of two papers showing an increase in basal rotation [32,56] and two showing no difference [21,31]. Of the two articles to show no difference however, one showed a non-significant increasing trend in HCM [21] and the other used a lesser known offline analysis software (velocity vector imaging) [31] which has shown to be only moderately correlated to basal rotation values derived through speckle tracking [57]. As such, we feel the limited available evidence leans towards an increase in basal rotation in those with HCM.

A total of nine articles reported on LV twist in those with HCM Again, the same four papers were methodologically sound and comparable, two of which showed no change in twist, while the same two articles to show no change in basal rotation reported no change in twist [32,56]. We again feel the difference could be due to image analysis techniques or possibly subtle differences in sample characteristics such as the ratio of obstructive to non-obstructive HCM patients. It should be noted that Carasso and colleagues used the same sample for both published articles in this section.

### *Diastolic parameters*

Diastolic LV rotation was shown to be impaired in those with HCM through consistent reports of decreased early untwist rate [32,55,56]. Two of these articles specifically reported a reduced percentage of untwist occurring during early diastole (5%, 10% and 15% of diastole) in those with HCM (Online Figure 4) [32,56]. Also, two articles described significant reductions in peak untwisting velocity in those with HCM [55,56], while one article with only seven participants showed a non-significant decrease [33]. Finally, average untwist rate was shown to not be different in those with HCM [33,35].

**Figure 4:**
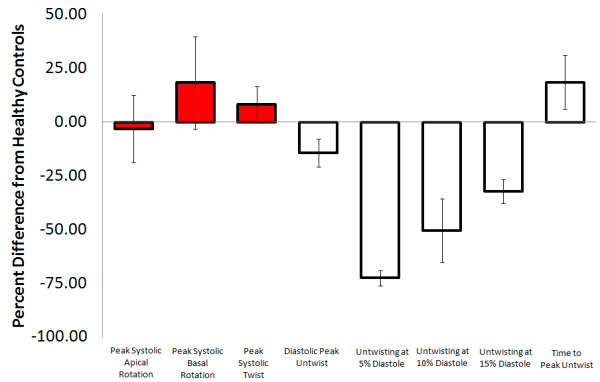
**Hypertrophic cardiomyopathy - **Average percent difference in left ventricular peak systolic apical rotation (Range: Control; 3.6 to 19.5, Patients; 4.1 to 12 degrees),[21,31-33,56] peak systolic basal rotation (Range: Control; -3.4 to −8.1, Patients; -3.2 to −6.6 degrees),[21,31-33,56] peak systolic twist (Range: Control; 6.6 to 22.6, Patients; 7 to 20 degrees)[21,23,32,33,55,56], untwisting at 5% diastole (Range: Control; 17 to 21, Patients; 10 to 12 percent),[32,56] untwisting at 10% diastole (Range: Control; 35 to 37, Patients; 23 to 25 percent),[32,56] untwisting at 15% diastole (Range: Control; 49 to 50, Patients; 36 to 39 percent)[32,56] and time to peak untwist (Range: Control; 14.6 to 111, Patients; 22.8 to 153% of systole (lower values normalized for diastolic duration)[55,56] between those with hypertrophic cardiomyopathy and healthy controls as reported in existing articles . Diastolic parameters denoted by empty boxes. Note that one author used the same population in two publications.[31,54] Therefore only one article was used in the percentage difference calculations.[31] Systolic parameters denoted by red filled boxes. Diastolic parameters denoted by empty boxes.

#### Conclusion*s*

There is substantial disagreement within the literature examining systolic LV rotation in HCM. It is likely that differences in methodological techniques as well as subtle differences between study populations are the cause of variability in this section, given the extremely heterogeneous nature of HCM phenotypic expression. It appears the disagreement is not due to methodological strength, as even the four strongest articles from this section reported opposite LV twist findings. With such substantial disagreement regarding LV rotation in those with HCM we feel it is not possible, until more work is completed, to comment on overall trends arising from the literature. Diastolic parameters of LV rotation were consistently shown to be impaired in those with HCM.

### Dilated cardiomyopathy

Nine articles examined LV rotation in those with dilated cardiomyopathy as compared to healthy controls [7,30,34-36,58-61]. Down and Black scores ranged from 15–19 (moderate methodological quality).

### *Systolic parameters*

Nine articles examined systolic LV rotation in those with dilated cardiomyopathy [7,30,34-36,58-61]. All articles to report these parameters were in agreement that apical rotation, LV twist [7,30,34-36,58-61] and torsion [7,30,34,61] were reduced (Online Figure 5). Of the nine papers that reported systolic basal rotation, seven showed a reduction while two reported no difference in those with dilated cardiomyopathy. Both articles that did not show a significant difference in basal rotation were completed by the same author, contained relatively small sample sizes (n = 10) and included only participants who had *restrictive* LV filling (not a criteria in the other articles) [36,60]. Of the three articles that reported on time to peak twist, two showed a significant increase in duration in the dilated cardiomyopathy group [58,61] while one paper showed no difference [36]. This latter article was one of the articles with modified inclusion criteria [36]. Lastly, it was consistently reported that large proportions of those with dilated cardiomyopathy have reverse rotation in either the apex or base [7,30,58].

**Figure 5:**
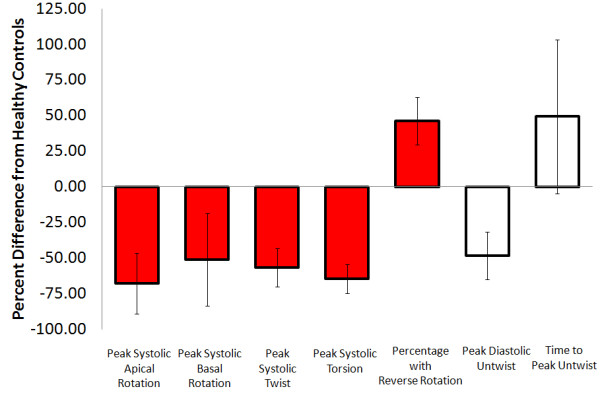
**Dilated cardiomyopathy -** Average percent difference in left ventricular peak systolic apical rotation (Range: Control; 5.4 to 15.8, Patients; 0.1 to 5.9 degrees),[7,30,34,36,58-61] peak systolic basal rotation (Range: Control; -2.6 to −7.1, Patients; -3.2 to −6.6 degrees),[7,30,34,36,58-61] peak systolic twist (Range: Control; 9.8 to 17, Patients; 4 to 7.35 degrees),[7,34,36,58-61] peak systolic torsion (Range: Control; 1.7 to 3, Patients; 0.4 to 1.3 degrees/cm),[7,34] percentage with reverse rotation,[7,30,58] peak diastolic untwist (Range: Control; -86 to −113, Patients; -37 to −62 degrees) [34,35,59,60] and time to peak untwist [59,60] between those with dilated cardiomyopathy and healthy controls as reported in existing articles. Systolic parameters denoted by red filled boxes. Diastolic parameters denoted by empty boxes.

### *Diastolic parameters*

All articles which reported on diastolic LV rotational parameters in those with dilated cardiomyopathy were in agreement that average and peak untwisting velocity was significantly decreased [34,35,59,60], while time to peak untwist was increased [59,60].

#### Conclusions

There is broad agreement, according to level three evidence (somewhat reliable), that systolic and diastolic ventricular rotation characteristics are reduced in those with dilated cardiomyopathy. Also there was agreement in all three articles where it was reported, implying that the LV rotates similar to a rotating pipe, instead of twisting sponge, in those with dilated cardiomyopathy.

### Non-compaction

Two articles investigated LV rotational parameters in those with non-compaction cardiomyopathy as compared to healthy controls and received D&B scores of 17 [36] and 19 [62] (moderate methodological quality).

### *Systolic parameters*

Bellavia and colleagues reported a reduction in apical rotation and twist as well as twist rate in a grouped sample of non-compaction participants [62]. When looking at a subgroup of non-compaction with normal EF, basal rotation was not different, but in a reduced EF (<50%) subgroup basal rotation was reduced [62]. Work by van Dalen also reported a reduction in apical rotation and LV twist in those with non-compaction [36]. Interestingly, van Dalen reported that rotation in all those with non-compaction was in the same direction at the apex and base, instead of opposite directions as in healthy controls. Roughly half non-compaction participants reported LV rotation in the counterclockwise direction at both the apex and base, while the other half reported clockwise rotation [36].

### *Diastolic parameters*: None reported

#### Conclusions

There is level three evidence (somewhat reliable) that systolic LV rotational parameters are reduced in those with non-compaction cardiomyopathy [36,62]. The evidence suggests that the LV rotates in unison at the base and apex resulting in very little twist [36]. A figure was not created for non-compaction, as groups were not comparable between articles.

### Systolic heart failure

Five articles investigated LV rotation in those with SHF as compared to matched controls [20,43,63-65]. Heart failure was diagnosed according to standards from the New York Heart Association (class III or IV) [43,63,64] or the Heart Failure and Echocardiography Associations of the European Society of Cardiology [20], while one article did not report specific criteria (however did report an EF of 26% in their clinical population) [65]. Downs and Black scores ranged from 17–23 (moderate to strong methodological quality).

### *Systolic parameters*

The three articles that reported on systolic apical and basal rotation were in agreement showing a reduction in both regions in SHF [43,64,65] (Online Figure 6). Similarly, all articles that reported on LV twist in those with systolic heart failure showed significant reductions [20,43,63,65]. Finally, one article reported that 59% of SHF patients had reversed rotation at either the basal or apical level [63].

**Figure 6:**
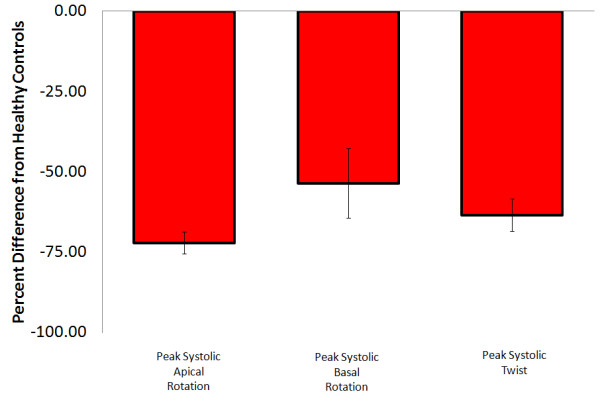
**Heart failure -** Average percent difference in left ventricular peak systolic apical rotation (Range: Control; 3.3 to 9.4, Patients; 1 to 2.4 degrees),[43,64,65] peak systolic basal rotation (Range: Control; -6.1 to −9, Patients; -3.3 to −3.5 degrees) [43,64,65] and peak systolic twist (Range: Control; 14 to 16.2, Patients; 4.8 to 6.8 degrees) [20,63,64] between those with heart failure and healthy controls as reported in existing articles. Systolic parameters denoted by red filled boxes.

### *Diastolic parameters*

The one article to report on LV diastolic rotation parameters in those with SHF showed apical untwisting was reduced while basal untwisting was not different [65].

#### Conclusions

There is level three evidence (somewhat reliable) that systolic LV rotation is altered in those with SHF. There is also level three evidence (somewhat reliable) that diastolic apical untwisting is altered in those with SHF. It appears that apical and basal rotation occurs in unison in a high proportion of those with SHF. More work is needed to clarify diastolic rotational motion of the LV in those with SHF.

### Diastolic Dysfunction/Heart Failure Normal Ejection Fraction

Heart failure normal ejection (HFnEF) fraction describes a significant reduction in LV filling during diastole (diastolic dysfunction), with a preserved EF. Diastolic dysfunction is described by four categories of increasing intensity, with HFnEF considered grades three and four as long as EF is preserved [66]. Left ventricle rotation in those with diastolic dysfunction as compared to healthy controls were examined by six published articles [3,20,37,67-69]. Downs and Black scores ranged from 17–23 (moderate to strong methodological quality).

### *Systolic parameters*

The five articles to report on systolic LV rotation in those with diastolic dysfunction have considerable disagreement between studies. Work by Park and colleagues reported a significant increase in apical rotation, basal rotation, twist and twist rate in those with grade one diastolic dysfunction (impaired relaxation), while those with more severe diastolic dysfunction were not different from controls [3]. Similarly, Mizuguchi et al. reported a reduction in twist rate as well as a trend toward reduced twist in participants with mild diastolic dysfunction (impaired relaxation) [37]. At first glance, these findings appear to be in opposition to the other articles which reported no difference in LV rotation between groups [20,67,68].

These disagreements can likely be explained by systolic LV rotation differing across the spectrum of diastolic dysfunction, as EF was preserved in all articles. Park and colleagues as well as Mizuguchi et al. only reported significant differences between the control group and those with the mildest form of diastolic dysfunction (impaired relaxation) whereas the two more severe categories were not different from those of controls regarding systolic LV rotation. In support of this contention, the only other article to look at an intermediate diastolic dysfunction group showed borderline significant increases in apical rotation (*P* = 0.07) and twist (*P* = 0.18), with no difference in the more severe diastolic dysfunction group.[68] Considering the available evidence, the literature supports the notion that systolic rotation is increased in those with mild diastolic dysfunction but normalizes in more severe stages of disease.

### *Diastolic parameters*

A relatively small number of articles reported diastolic LV rotation in those with diastolic dysfunction.[3,67,68] Park and colleagues showed that untwist rate was increased in those with grade one diastolic dysfunction. Also two articles reported that those with moderate diastolic dysfunction, untwist rate [3] and time to peak untwist [67] were not different than those of controls. Further, Park et al. showed that those with grade three diastolic dysfunction had untwisting rates less than those found in healthy controls. Finally, Perry et al. showed that peak early diastolic apical untwist was reduced further with increases in grade of diastolic function [69]. Again, it appears from the literature that diastolic LV rotation is increased in those with moderate diastolic dysfunction but reduces as severity of disease increases.

#### Conclusions

There is level three evidence (somewhat reliable) that LV rotation in both systole and diastole is increased in those with mild diastolic dysfunction (Online Figure 7). Further to this, there is level three evidence (somewhat reliable) to suggest that LV systolic rotation is not different from health controls in more severe stages of disease [3,37,68]. More work is needed to clarify diastolic LV rotation in different stages of diastolic dysfunction however it appears that diastolic rotation is increased in mild, similar in moderate and reduced in severe diastolic dysfunction as compared to healthy controls.

**Figure 7:**
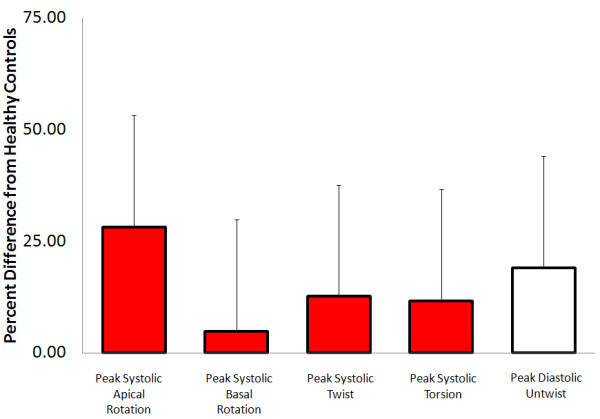
**Diastolic dysfunction/heart failure normal ejection fraction.** Average percent difference in left ventricular peak systolic apical rotation (Range: Control; 7.8 to 9.9, Patients; -8.4 to 15.7 degrees), [3,67,68] peak systolic basal rotation (Range: Control; -6.3 to −8, Patients; -7.1 to −8.2 degrees), [3,67,68] peak systolic twist (Range: Control; 14 to 15.8, Patients; 13 to 16.9 degrees), [3,20,37,68] peak systolic torsion (Range: Control; 2.2 to 2.5, Patients; 2.5 to 2.7 degrees/cm) [37,67] and peak diastolic untwist (Range: Control; -110 to −112, Patients; -129 to −135 degrees/sec) [3,67] between a pooled sample of those with any grade of diastolic dysfunction and healthy controls as reported in existing articles. Systolic parameters denoted by red filled boxes. Diastolic parameters denoted by empty boxes.

### Combined categories

Several heart diseases have been investigated by a single article and are discussed here in unison in the interest of readability. The individual ranking and other specific details for each article can be reviewed in Table 7 (Online). Pacemaker implantation [70], and constrictive pericarditis [29] all had reduced systolic rotation while those with heart transplants [5], restrictive cardiomyopathy [29] and cardiovascular risk factors [37] did not. The only article from this group to report diastolic parameters showed no difference between heart transplant recipients and healthy recipient-aged and donor-aged controls. Interestingly, heart transplant recipients did have significantly reduced twist and peak untwist rate during exercise but not at rest [5]. Paetsch et al. examined LV rotation in patients with coronary artery disease showing apical systolic rotation and diastolic apical untwisting was reduced at both high and low dobutamine doses while time to peak untwist was reduced in comparison to healthy controls in the low dose only [71].

## Discussion

The purpose of this review was to compile and evaluate relevant literature examining the difference between those with various heart diseases and healthy individuals with regard to LV rotation. Due to ethical limitations to inducing disease in the human model, study design throughout the review is almost entirely comprised of level three evidence. Although this limitation is valid, a large cohort study design that follows participants from the relatively healthy early years of life, to later life when heart disease is apparent, would provide level two evidence, and aid in the understanding of the sequence of LV rotation abnormalities in various heart disease states. Unfortunately, it is outside the scope of this systematic review to comprehensively discuss underling mechanisms responsible for each change in LV rotation although brief summaries have been provided in each conclusion paragraph.

Taking into consideration the available evidence (heart diseases with more than one published article which contain quantified numeric outcome values), it appears that aortic stenosis as well as SHF and dilated cardiomyopathy lead to the most profound changes in LV systolic rotation as compared to healthy controls. According to the same criteria, in diastole, MI, HCM and dilated cardiomyopathy have the largest reductions in LV rotational parameters. Interestingly, aortic stenosis has considerable support showing systolic rotation is increased as compared to healthy controls. It is also worth note that there are no trends in the literature suggesting systolic-diastolic rotational uncoupling in heart disease.

In an effort to provide clinical meaning, it appears from the literature that heart disease with heterogeneous characterization such as MI and HCM show less agreement in relationship to parameters of LV rotation. To our knowledge no literature exists regarding the prognostic value of LV rotation. In light of the reports found in this article, there may be clinical value in monitoring LV rotation in those at the highest risk for aortic stenosis with no other clinical markers of LV dysfunction, as well as untwist in those with suspected diastolic dysfunction.

Several studies did not utilize relevant control groups, matched on known cardiovascular confounding factors such as age (See Tables 2, 3, 4, 5, 6, 7, 8 for specific details). This shortcoming is most apparent in articles investigating heart diseases occurring late in life, possibly due to the perceived relative difficulty in recruiting control volunteers in their mid sixties as compared to those in a younger age group. We encourage authors to be diligent in regard to appropriate matching of controls. If matched-groups are not possible, statistical procedures such as analysis of co-variance (ANCOVA) should be employed to better evaluate the independent effect of the specific heart disease in question. We did find however, that other important confounders such as gender, blood pressure, LV mass, LV volume, and EF were relatively well matched or accounted for throughout. Another common shortcoming in the literature is the widespread omittance of data reporting the duration of time participants have been diagnosed with a given disease, which would improve our understanding of the pathological sequences.

There is considerable variability in the context of terminology used to describe LV rotation between journal articles. For example, torsion is commonly used to define twist and vice versa, while twist in some articles is instead used to describe basal or apical rotation. Moreover, several different measures of diastolic rotation are employed throughout the literature. Most commonly, untwist rate is reported however some authors prefer early untwist rate, untwist rate at 5%, 10%, and 25% of diastole, while still others report apical untwisting rate. Taking care to standardize parameters in this relatively new practice of measuring LV rotation may increase the rate at which the field progresses, by allowing similar studies to directly compare results.

Finally, exercise has been shown to be a powerful tool for increasing the sensitivity of tests designed to diagnose LV functional changes. In light of this we feel further studies should employ, when possible, an exercise stimulus while examining differences in LV function in those with heart disease. Where it has been reported, exercise has exaggerated differences in LV rotation between healthy and diseased hearts [5,25]. The evaluation of LV rotational motion has been studied during exercise with relative success at sub-maximal exercise intensities [72].

Several limitations exist in this systematic review which we have made efforts to mitigate through our study design. Firstly, there is evidence that significant variability exists between and within and between speckle-tracking software [73]. We have attempted to control for this in our summary figures by calculating the percent difference between controls and heart disease groups for each article, and averaging the results. This will at least ensure that differences in absolute values are not compared between articles which may not have comparable absolute results. Detailed recommendations for moving forward in this line of research to ensure comparability between studies have been recently published [73]. It is well known that heart diseases are not mutually exclusive and often more than two or more heart diseases are present in a given patient. For this reason we acknowledge that many of the subjects included in the reviewed articles may have had other heart diseases and significant overlap between values may have occurred. In any case, we feel the overall findings do shed light on trends in LV rotational changes occurring in a group with a common principle heart disease. Also, although the vast majority of articles imaged apical and basal rotation according to the standard landmarks, there may be some variability between studies which employed different short axis levels of apical rotation; resulting in more mid-level oriented images. We have tried to account for this by reporting the short axis images collected by each study in the comprehensive tables (Online Supplement Tables 3,4,5,6,7,8,9). As a final point, it should be mentioned that one article has shown three-dimensional STE to be more sensitive to changes in LV rotation occurring due to heart disease [74]. Additional articles on this topic are required to corroborate this finding but an early assumption would be that 2D-STE may underestimate some measures of LV rotation in those with heart disease.

## Conclusions

Left ventricular rotation parameters in those with various heart diseases are not commonly investigated in comparison to healthy controls but can add important insight into LV functional changes occurring during heart disease progression. This is likely due to the novel and time-consuming nature of the measurement techniques. Specifically, heart transplant, pacemaker implantation and pericardial abnormalities have a glaring shortage of available literature comparing with healthy controls. According to the available literature, LV rotation in both systole and diastole are altered in various forms of heart disease. The various parameters commonly measured (i.e., apical rotation, untwist rate) appear to follow their own disease-dependent pattern.

## Competing interests

The authors declare that they have no competing interest.

## Authors’ contributions

AAP conceptualized the topic, performed the literature search, drafted the manuscript and edited the manuscript. ATC independently performed the literature search and edited the manuscript. SSDB conceptualized the topic and edited the manuscript. DERW conceptualized the topic and edited the manuscript. All authors read and approved the final manuscript.

## Funding

This research was supported by funding from the Canadian Institutes of Health Research, the Michael Smith Foundation for Health Research, the Natural Sciences and Engineering Research Council of Canada, the Canada Foundation for Innovation and the BC Knowledge Development Fund. AA Phillips was supported by funding from Natural Sciences and Engineering Research Council and the Mathematics of Information and Complex Systems. DER Warburton was supported by salary awards from the Canadian Institutes of Health Research and the Michael Smith Foundation for Health Research.

## Pre-publication history

The pre-publication history for this paper can be accessed here:

http://www.biomedcentral.com/1471-2261/12/46/prepub
